# Hemostatic changes after 1 month of thalidomide and dexamethasone therapy in patients with multiple myeloma

**DOI:** 10.1007/s12032-012-0290-0

**Published:** 2012-07-07

**Authors:** Marta Robak, Jacek Treliński, Krzysztof Chojnowski

**Affiliations:** Department of Hematology, Copernicus Memorial Hospital, Medical University of Lodz, ul Ciolkowskiego 2, 93-510 Lodz, Poland

**Keywords:** Multiple myeloma, Platelet activation, Hypercoagulation, Thalidomide, Dexamethasone, Thrombosis

## Abstract

Thromboembolic events (TEE) are a serious clinical problem in multiple myeloma (MM) patients receiving thalidomide (T). Thirty-one MM patients were tested on diagnosis and after 2 and 4 weeks of therapy with T alone, or T in combination with dexamethasone (TD). Closure time (CT) in PFA-100 and P-selectin expression were assessed, as well as plasma levels of thrombin-antithrombin complexes (TAT), D-dimer (DD), soluble thrombomodulin (sTM) and von Willebrand factor antigen (vWF:Ag), along with the activity of coagulation factor VII and factor VIII. The concentration of vascular endothelial growth factor and its type 1 and 2 receptors were also assayed. On diagnosis, significantly prolonged median CT with both used cartridges, elevated P-selectin expression, DD concentration, TAT, vWF:Ag and factor VIII and factor VII activity were seen in the patient group as compared to controls. Therapy with these regimens caused marked shortening of CT with both cartridges. Treatment with TD leads to the significant increase in CD62P expression on platelets. Median TAT value increased significantly in relation to baseline after therapy with both regimens. Factor VIII activity exceeded 150 % in all patients after 2 weeks of TD therapy and was markedly elevated compared to baseline. One month of TD therapy significantly increased sTM concentration. These results demonstrate the enhanced platelet and coagulation system activation already present in MM patients on diagnosis, which is further increased by antimyeloma therapy. These changes are more pronounced after TD therapy and may promote TEE. Tested angiogenesis marker levels are elevated already on diagnosis, do not change after therapy and have no significant impact on the coagulation system in patients with MM.

## Introduction

Different abnormalities of hemostasis, resulting in bleeding or thromboembolic events (TEE), are a serious clinical problem in patients with multiple myeloma (MM). These complications are due to acquired platelet and coagulation system defects, but their exact pathogenesis remains unexplained. The introduction of new antiangiogenic drugs resulted in an increase in both the overall survival rates and TEE frequency in this group of patients. The incidence of thrombosis is reported to be about 2–5 % for thalidomide (T) in monotherapy and 10–30 % when thalidomide is combined with dexamethasone (TD) [1–3].

Some reports confirm the benefits of low-dose aspirin in preventing these complications, suggesting that platelet activation may be an important pathogenic factor [4–8]. However, until now, platelet activation in MM patients treated with T and TD has not been studied in detail. Also, the influence of these agents on activation of the coagulation system is yet to be fully elucidated. In recent years, it has been demonstrated that vascular endothelial growth factor (VEGF) may be involved in coagulation through interactions with tissue factor (TF). VEGF enhances the expression of TF on monocytes and endothelial cells, thus triggering the coagulation cascade [9, 10]. The aim of the present study was to assess certain aspects of platelet activation and coagulation activation, as well as angiogenesis markers in patients with multiple myeloma: firstly on diagnosis and then in relation to antimyeloma therapy.

## Patients and methods

The study was performed on 31 MM patients with a median age of 66.1 years (range 42–83 years) (Table 1) after giving informed consent. MM IgG was diagnosed in 22 patients, MM IgA in 7 and light chain disease in 2. All patients were in stage II or III of disease according to the Durie and Salmon classification. Only patients with a platelet count PLT > 100 × 10^9^/l were enrolled onto the study. The exclusion criteria were as follows: renal failure (creatinine level ≥ 2 mg/dl), diabetes, history of thrombosis, surgery in the past 3 weeks or administration of drugs strongly influencing platelet function 10 days prior to study entry.

**Table 1:** Characteristics of multiple myeloma patients

Type of MM	Gender	Age (years) [median (range)]	Durie and Salmon (II/III)
	Female/men		
MM IgG	13/9	67.9 (47–76)	5/17
MM IgA	3/4	64.0 (42–83)	2/5
LCD	0/2	54.0 (48–60)	0/2
Total	16/15	66.1 (42–83)	7/24

*MM* multiple myeloma, *LCD* light chain disease

The tests were performed at the time of diagnosis and than repeated after two and 4 weeks of treatment. Sixteen patients were treated with T (100–200 mg/day po), and fifteen with T in combination with dexamethasone (20 mg/day or 40 mg/day in days 1–4, 9–12, 17–20). The groups did not differ from each other markedly in relation to age, platelet count, hemoglobin concentration, monoclonal protein concentration or clinical stage of the disease. The control group consisted of 20 healthy subjects (14 women and 6 men) at similar age.

In each patient, basic tests of hemostasis and closure time (CT) were screened with collagen/ADP (Col/ADP) and collagen/epinephrine (Col/Epi) cartridges in a PFA-100 platelet function analyzer (Dade Behring, Newark DE). Platelet activation was estimated by P-selectin expression (fluorescein isothiocyanate conjugated CD62P, Serotec, Oxford, UK) in flow cytometry. Additionally, the plasma concentration of thrombin-antithrombin complexes (TAT) (ELISA kit, Siemens Healthcare Diagnostic), D-dimer (DD) (Immunoturbidimetric method, Diagnostica Stago, France), soluble thrombomodulin (sTM) (Elisa kit, Diaclone, France), vWF:Ag (Diagnostica Stago, France) and activity of coagulation factor VII, factor VIII were evaluated (Diagnostica Stago, France). Furthermore, in 25 patients, such angiogenic factors as VEGF and soluble vascular endothelial growth factor receptors 1 and 2 (sVEGFR-1, sVEGFR-2) were assayed by specific, commercially available, ELISA kits (Human VEGF, sVEGF R1/Flt-1, sVEGF R2/KDR Quantikine ELISA, R&D Systems Inc., USA).

### Statistical analysis

Data are presented as medians and ranges for continuous variables, and as mean and standard deviation for categorical variables. Statistical comparisons between groups were made by the Student *t* test or Mann–Whitney *U* test. The Wilcoxon test was used to compare the results of patients before and after therapy with T or TD. Correlations between variables were assessed by the Spearman rank correlation coefficient (*r*). In all tests, *p* values less than 0.05 were considered statistically significant. Statistical analysis was performed using STATISTICA v.s.8.0 (Tulsa, OK, USA) software.

## Results

### Selected primary hemostasis parameters

The data concerning the primary hemostasis tests are presented in Table 2. At the time of diagnosis, the mean CT was significantly prolonged in the patient group when compared to controls (Col/ADP 105.5 s vs. 88.0 s; *p* = 0.002, Col/Epi 143.0 s vs. 109 s; *p* = 0.001). A significant correlation between IgG concentration and CT was observed with the Col/ADP cartridge (*r* = 0.52; *p* < 0.001) and with the Col/Epi cartridge (*r* = 0.51; *p* < 0.001).

**Table 2:** Results of selected primary hemostasis parameters before and after treatment

Parameter	Before treatment A	After 2 weeks of treatment B	After 4 weeks of treatment C	Control D	*p*
CT Col/ADP (s)					A&B (*p* = 0.09)
All patients	105.5	99.0	89.0	88.0	A&C (*p* < **0.001**)
	63–255	57.0–202.0	56–161	70–108	A&D **(** ***p*** = **0.002)**
Treated with T	104.5	105.0	87.5		A&B (*p* = 0.75)
	63–225	74–202	58–123		A&C (***p*** = **0.04**)
Treated with TD	113.0	91.5	89.0		A&B (***p*** = **0.003**)
	80–176	57–136	56–161		A&C (***p*** **<** **0.001**)
CT Col/Epi (s)					A&B (*p* = 0.15)
All patients	143.0	136.0	114.0	109.0	A&C (***p*** **<** **0.001**)
	98–288	80.0–234.0	79–187	88–158	A&D (***p*** **=** **0.001**)
Treated with T	139.0	141.0	126.5		A&B (*p* = 0.40)
	98–288	80–234	79–187		A&C (***p*** **=** **0.01**)
Treated with TD	145.0	132.0	110.0		A&B (*p* = 0.18)
	100–182	81–174	90–161		A&C (***p*** = **0.009**)
CD62P (%)					A&B (*p* = 0.2)
All patients	3.1	2.9	3.9	0.74	A&C (***p*** **=** **0.02**)
	0.1–21.5	0.4–19.3	0.8–22.2	0.11–2.35	A&D (***p*** **<** **0.001**)
Treated with T	2.3	2.3	3.4		A&B (*p* = 0.97)
	0.1–21.5	0.4–5.4	0.8–10.9		A&C (*p* = 0.05)
Treated with TD	3.6	4.2	4.5		A&B (*p* = 0.06)
	0.6–9.7	0.6–19.3	1.6–22.2		A&C (***p*** **=** **0.04**)

The data is reported as median and range

*T* thalidomide, *TD* thalidomide and dexamethasone

Bold values are statistically significant at *p* < 0.05

Therapy with T resulted in marked shortening of CT with Col/ADP (104.5 vs. 87.5; *p* = 0.04) and with Col/Epi (139 vs. 126.5; *p* = 0.01) after 4 weeks of treatment. TD therapy was additionally associated with significant shortening of CT with Col/ADP after 2 weeks of treatment (113 vs. 91.5; *p* = 0.003).

Significantly elevated expression of P-selectin (CD62P) on platelets (3.1 vs. 0.74 %; *p* < 0.001) was seen in the MM group in relation to controls. Four weeks of thalidomide therapy resulted in no significant change in the percentage of platelet CD62P expression, while therapy with TD resulted in a marked increase (3.6 vs. 4.5 %; *p* = 0.04).

### Selected coagulation parameters

The median values of coagulation activation markers (DD, TAT) on diagnosis were significantly increased compared to the controls (Table 3). Markedly increased TAT values were seen in relation to baseline after 4 weeks’ therapy with both treatment regimens. The median values of factor VII, factor VIII and vWF:Ag were significantly higher in the patient group compared to controls. Factor VII demonstrated markedly lower median activity after 1 month of TD treatment, while factor VIII demonstrated higher activity (>150 % in all patients) after 2 weeks of TD therapy. A positive correlation between factor VIII activity and TAT concentration (*r* = 0.34; *p* = 0.004) was found.

**Table 3:** Results of selected coagulation parameters before and after treatment

Parameter	Before treatment A	After 2 weeks of treatment B	After 4 weeks of treatment C	Control D	*p*
TAT (μg/l)					A&B (*p* = 0.3)
All patients	4.1	3.8	5.5	2.0	A&C (***p*** **=** **0.003)**
	2.0–14.5	1.4–13.8	2.2–42.8	1.3–2.7	A&D (***p*** **<** **0.001)**
Treated with T	4.0	4.3	6.1		A&B (*p* = 0.22)
	2.0–14.5	1.6–12.5	2.2–43.8		A&C (***p*** **=** **0.004)**
Treated with TD	4.4	3.8	5.9		A&B (*p* = 0.78)
	2.0–6.9	1.4–13.8	2.4–33.7		A&C (***p*** **=** **0.02)**
D-dimer (μg/ml)					A&B (*p* = 0.94)
All patients	1.5	1.2	2.3	0.2	A&C (***p*** = **0.04)**
	0.22–6.03	0.26–8.25	0.22–17.4	0.14–0.41	A&D (***p*** **<** **0.001)**
Treated with T	2.0	1.3	2.3		A&B (*p* = 0.65)
	0.4–6.0	0.4–8.2	0.22–15.1		A&C (*p* = 0.14)
Treated with TD	1.0	1.1	0.95		A&B (*p* = 0.47)
	0.2–5.1	0.26–4.6	0.27–17.4		A&C (*p* = 0.27)
Fibrinogen (mg/dl)					A&B (*p* = 0.6)
All patients	397.0	379.0	429.5	270.0	A&C (***p*** **=** **0.02)**
	146–647	157–692	220–818	246–307	A&D (***p*** **<** **0.001)**
Treated with T	397.5	426.5	441.0		A&B (*p* = 0.80)
	249–637	157–692	224–780		A&C (*p* = 0.08)
Treated with TD	383.0	374.5	418.0		A&B (*p* = 0.27)
	146–647	222–668	220–818		A&C (*p* = 0.10)
FVII (%)					A&B (*p* **=** 0.09)
All patients	109.0	90.5	91.5	98.0	A&C (***p*** = **0.003**)
	47–156	49–181	26–133	84–103	A&D (*p* = 0.14)
Treated with T	105.0	89.0	82.0		A&B (*p* = 0.33)
	55–125	58–145	52–133		A&C (*p* = 0.06)
Treated with TD	123.0	100.0	101.5		A&B (*p* = 0.17)
	47–156	49–181	26–124		A&C (***p*** **=** **0.02**)
FVIII (%)					A&B (*p* = 0.07)
All patients	235.0	230.0	242.0	84.0	A&C (*p* = 0.15)
	80–381	138–600	135–558	76–95	A&D (***p*** **<** **0.001)**
Treated with T	226.0	209.5	238.0		A&B (*p* = 0.92)
	96–420	138–320	135–558		A&C (*p* = 0.07)
Treated with TD	254.5	316.5	247.0		A&B (***p*** = **0.03**)
	80–381	184–600	143–447		A&C (*p* = 0.69)
vWF:Ag (%)					A&B (*p* = 0.61)
All patients	171.0	160.0	166.0	91.5	A&C (*p* = 0.94)
	54–420	41–320	58–337	81–107	A&D (***p*** **<** **0.001**)
Treated with T	166.0	172.7	178.0		A&B (*p* = 0.87)
	106–283	119–320	124–289		A&C (*p* = 0.99)
Treated with TD	173.5	156.0	165.0		A&B (*p* = 0.51)
	54–323	41–199	58–337		A&C (*p* = 0.97)
sTM (ng/ml)					A&B (*p* = 0.65)
All patients	3.2	3.8	4.0	2.9	A&C (***p*** **=** **0.03**)
	1.5–7.4	1.8–10.8	1.9–10.3	2.2–4.9	A&D (*p* = 0.14)
Treated with T	3.2	3.5	4.0		A&B (*p* = 0.47)
	2.2–7.4	1.8–10.8	2.2–8.9		A&C (*p* = 0.75)
Treated with TD	3.3	4.1	4.2		A&B (*p* = 0.07)
	1.5–6.2	2.0–6.5	1.9–10.3		A&C (***p*** = **0.006**)

Data is reported as median and range

*T* thalidomide, *TD* thalidomide and dexamethasone

Bold values are statistically significant at *p* < 0.05

At the time of diagnosis, the sTM concentration values did not differ significantly between patients and controls. However, after 1 month of TD therapy, a markedly increased sTM concentration was observed (3.3 ng/ml vs. 4.2 ng/ml; *p* = 0,006). Additionally, at that time, a significant positive correlation was found between sTM concentration and vWF:Ag (*r* = 0.34, *p* = 0.02), as well as between sTM and D-dimer concentration (*r* = 0.36, *p* = 0.01).

### Selected angiogenesis parameters

Significantly higher median concentrations of VEGF and sVEGFR1 were seen in the MM group in relation to control. Although the median levels of VEGF, sVEGFR1 did not differ, sVEGFR2 was markedly elevated after 1 month of TD therapy (Table 4). No marked correlations were found between these angiogenic factors and markers of coagulation and fibrinolysis activation.

**Table 4:** Results of selected angiogenesis parameters before and after treatment

Parameter	Before treatment A	After 2 weeks of treatment B	After 4 weeks of treatment C	Control D	*p*
VEGF (pg/ml)					A&B (*p* = 0.44)
All patients	31.7	32.2	32.2	9.7	A&C (*p* = 0.73)
	9.7–112.5	8.5–98.3	7.9–64.7	0.6–20.7	A&D (***p*** **<** **0.001**)
Treated with T	24.4	30.5	30.5		A&B (*p* = 0.50)
	9.7–112.5	15.9–93.7	14.0–64.7		A&C (*p* = 0.80)
Treated with TD	34.1	37.1	33.9		A&B (*p* = 0.82)
	10.3–98.3	8.5–98.3	7.9–54.4		A&C (*p* = 0.47)
sVEGFR1 (pg/ml)					A&B (*p* = 0.59)
All patients	100.5	114.6	110.3	63.7	A&C (*p* = 0.88)
	59.7–265.3	66.8–243.3	61.6–257.2	49.6–75.0	A&D (***p*** **<** **0.001**)
Treated with T	100.5	105.4	109.8		
	59.7–265.3	66.8–243.3	61.6–257.9		
Treated with TD	103.2	119.2	111.4		A&B (*p* = 0.58)
	77.7–236.1	73.3–176.9	81.0–247.5		A&C (*p* = 0.21)
sVEGFR2 (pg/ml)					A&B (*p* = 0.56)
All patients	5,940	5,965	5,667	6,430	A&C (*p* = 0.12)
	4,020–8,815	3,975–10,400	3,930–9,520	3,795–7,540	A&D (*p* = 0.10)
Treated with T	5,940	5,965	5,190		A&B (*p* = 0.13)
	4,100–5,881	3,975–8,530	3,930–9,125		A&C (*p* = 0.86)
Treated with TD	5,760	6,495	5,920		A&B (*p* = 0.12)
	4,020–7,540	4,045–10,400	4,590–9,520		A&C (***p*** **=** **0.03**)

Data is reported as median and range

*T* thalidomide, *TD* thalidomide and dexamethasone

Bold values are statistically significant at *p* < 0.05

## Discussion

To the best of our knowledge, this is the first study assessing CT in patients with MM after therapy with T and TD. We found that, at the time of diagnosis, the Col/ADP CT value was above the normal range in 33 % of the patients, and the Col/Epi CT value in 30 %. This was probably due to the influence of the monoclonal protein on platelet function, which was confirmed by the positive correlation between monoclonal IgG protein concentration and CT with Col/Epi. Therapy with both treatment modalities resulted in significant shortening of CT with both cartridges, possibly due to an improvement in platelet function caused by the reduction in monoclonal protein level found after antimyeloma therapy.

To date, there have been only a few studies concerning the influence of T on platelet activation in MM patients [5, 11]. In the present study, a markedly higher expression of P-selectin on platelets was found at diagnosis in patients with MM as compared to healthy volunteers. In addition, therapy with TD was seen to result in significant elevation of P-selectin in relation to baseline. Previously, Dunkley et al. [5] assessed P-selectin expression in five MM patients on diagnosis, after 1 month of T therapy and then after 2 weeks of aspirin treatment. P-selectin expression was first seen to be elevated after therapy with T, and later to decrease in connection with aspirin use. The authors suggest that T induces platelet activation that can be suppressed by aspirin use. Two of the most probable mechanisms of platelet activation are direct, though the influence of T on the platelets, and indirect, via activation of endothelial cells. Until now, there have been no reports concerning the influence of combined therapy with TD on platelet activation.

It has to be stressed that few good quality studies assessing the impact of corticosteroids on platelet function in varied clinical settings have been published. Jilma et al. [12] show that high-dose dexamethasone given to 9 healthy volunteers caused enhanced platelet activation, displayed by increased soluble P-selectin expression. These results are in agreement with our observations, although we estimated the combined action of dexamethasone and T.

The elevated activity of coagulation factor VIII and von Willebrand factor (vWF) is one of the possible mechanisms leading to thrombosis in MM patients. Minnema et al. [13] found that factor VIII activity >150 % is bound with a 5-times higher risk of developing thrombosis in relation to normal values. In our patient cohort, increased activities of factor VIII (>150 %) and vWF:Ag (>160 %) were observed on diagnosis in 78 and 66 % of patients, respectively. The median levels of these parameters were markedly higher than in controls, and a significant positive correlation between these parameters was discovered. Therapy with T leads to increased factor VIII activity but without any statistical significance (*p* = 0.07). However, treatment with TD resulted in marked, but transient, increase in factor VIII activity. This phenomenon may be one of the possible causes of TEE development in the early period of antimyeloma treatment. The fluctuation of factor VIII activity during TD therapy may also account for the discrepancies concerning FVIII behavior obtained by other authors [13–15]. Our observations are in partial agreement with findings by Minnema et al. [13] who observed elevated factor VIII activity and vWF:Ag in patients with MM treated with TD, or T, in combination with other chemotherapy regimens. The authors suggest that increased activity of these coagulation factors is more due to the activity of MM than to any adverse effects of therapy, since increased levels were found in all patients with active disease, no matter whether they had received treatment with T or not. The activity of factor VII diminished markedly during TD therapy when compared to baseline, which is difficult to interpret. One can not exclude that it can be related to the lowering of monoclonal protein due to antimyeloma therapy.

We found elevated levels of fibrinogen (>400 mg/dl) in 50 % of the patients already on diagnosis. Additionally, positive correlations between concentration of fibrinogen and D-dimer and factor VIII activity were seen. The median levels of fibrinogen concentration increased during therapy, the phenomenon that was also observed by others in the past [15, 16]. Our results indicate that increased fibrinogen concentration is mainly due to MM activity and is not further enhanced by therapy with T or TD. The presence of a positive correlation between fibrinogen concentration and D-dimer strongly suggests that it plays a role in the hypercoagulable state in MM.

The influence of T on coagulation activation markers has been studied by a number of authors [17–19]. We observed markedly higher concentrations of TAT and D-dimer in our MM patients on diagnosis in relation to controls. Therapy with T and TD leads to further increase in thrombin generation measured indirectly by TAT levels. This contradicts the findings of Streetly et al. [17] who found no increase in prothrombin fragment 1 + 2 in MM patients after a 28 day course of therapy with T analog.

Angiogenesis plays a crucial role in processes connected with the proliferation and growth of MM cells. VEGF and its receptors seem to be the most selective growth factor acting on endothelial cells and the major inducer of normal and pathological angiogenesis [20, 21]. Some authors suggest that VEGF has an important role in the activation of the coagulation system [9, 10]. VEGF increases TF expression on the surface of monocytes and endothelial cells, leading to enhanced platelet adhesion, activation and initiation of the coagulation system [9]. However, to date, there have been no studies concerning interactions between VEGF and coagulation activation markers in patients with MM in relation to T therapy. In our study, median levels of VEGF and sVEGFR1 were markedly higher than in controls, which is confirmed by results obtained by other authors [22–24].

Despite this, we did not observe any marked differences between the median levels of VEGF and sVEGFR1 after 2 and 4 weeks of therapy with both modalities. Although this is in agreement with findings by Neben et al. [25] who also did not find significant changes in VEGF after 6 months of T therapy, it contradicts the conclusions of Du et al. [24], Bertolini et al. [26] and Dmoszyńska et al. [27] who observed lower values of VEGF during T treatment. However, in the last two cases, the decrease in VEGF concentration concerned only patients with a good reaction to antimyeloma therapy. In another paper, Hatjiharissi et al. [23] suggests that these contradictory results depend on the dose of T used by particular investigators. In our study, no marked correlations were found between studied angiogenesis markers and either coagulation activation markers (TAT, D-dimer) or P-selectin expression. These results indicate that the influence of VEGF on TF expression has no direct impact on thrombin generation and platelet activation.

In conclusion it has been demonstrated that primary hemostasis abnormalities, estimated by the PFA-100 method, enhanced platelet activation, and hypercoagulable state are already present in MM patients at the time of diagnosis. Antimyeloma therapy of four weeks of causes a retreat of primary hemostasis defects (probably due to a decrease of monoclonal protein), increases platelet activation and enhances the hypercoagulable state in MM patients. Combined therapy with thalidomide and dexamethasone has a more pronounced influence on platelet activation and some aspects of hypercoagulability than treatment with thalidomide in monotherapy. VEGF and sVEGFR1 levels are elevated already on diagnosis, do not change after therapy with T or TD and have no impact on the activation of coagulation in patients with multiple myeloma.
